# Polysplenia syndrome complicated by multiple intrahepatic bile duct stones in an adult: a case report

**DOI:** 10.3389/fmed.2026.1704503

**Published:** 2026-02-17

**Authors:** Jiahao Zhang, Jiliang Lu, Hongzhong Liang, Pengyu Chen, Zheng Wang, Naiqing Liu, Jinghua Liu

**Affiliations:** 1 Department of General Surgery Center, Linyi People’s Hospital, Shandong Second Medical University, Linyi, China; 2 School of Clinical Medicine, Shandong Second Medical University, Weifang, China

**Keywords:** case report, hepatobiliary anomalies, imaging, multiple intrahepatic bile duct stones, polysplenia syndrome

## Abstract

Polysplenia syndrome is a rare congenital disorder characterized by multiple spleens and complex visceral and vascular anomalies. We report an adult case admitted for multiple intrahepatic bile duct stones. Imaging revealed multiple congenital abnormalities, including polysplenia, a truncated pancreas, and absence of the superior inferior vena cava segment. Intraoperative findings confirmed extensive intrahepatic bile duct stones and biliary malformations. Surgical treatment included partial hepatectomy, bile duct exploration, and T-tube drainage. Postoperatively, the patient developed bacteremia, which resolved with targeted antibiotics. This case highlights the link between polysplenia syndrome and hepatobiliary anomalies, emphasizing the need for careful anatomical evaluation, early recognition of biliary complications, and close clinical monitoring in affected patients.

## Introduction

Polysplenia syndrome is a rare congenital disorder characterized by the presence of multiple spleens and various visceral and vascular anomalies (1, 2). While the condition is frequently diagnosed in infancy due to severe cardiac malformations, survival into adulthood is uncommon (3). In adult cases, polysplenia syndrome is often discovered incidentally during imaging for unrelated clinical indications. Among the various anomalies associated with polysplenia syndrome, hepatobiliary malformations are of particular relevance in adults, as they may predispose to bile stasis, recurrent inflammation, and stone formation (4). However, reports of adult patients with polysplenia syndrome complicated by intrahepatic bile duct stones remain rare, with no standardized diagnostic or therapeutic approach established. Here, we report the case of a 60-years-old female who developed multiple intrahepatic bile duct stones with previously unrecognized polysplenia syndrome.

## Case presentation

**FIGURE 1:**
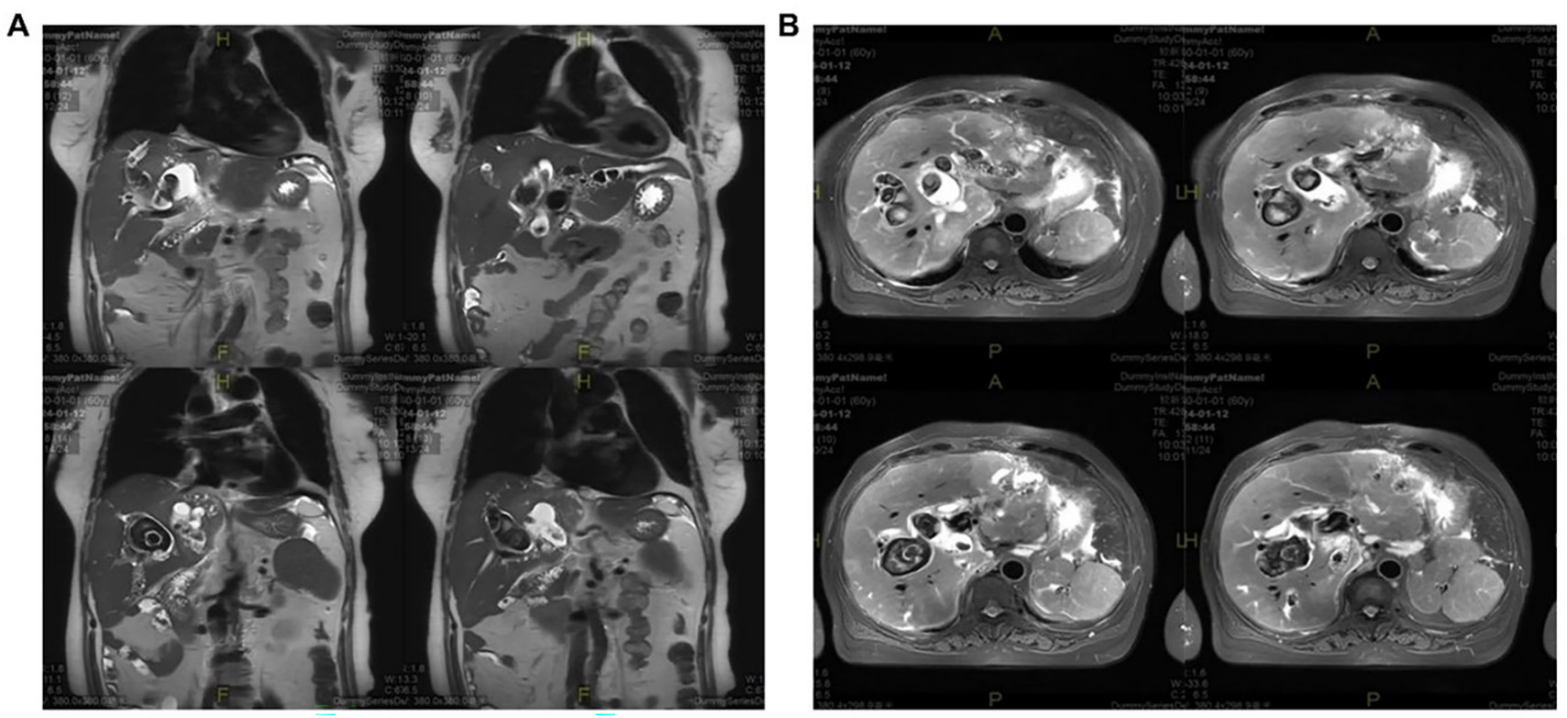
MRI images demonstrating multiple bile duct stones in panel **(A)** coronal and **(B)** axial planes.

**FIGURE 2:**
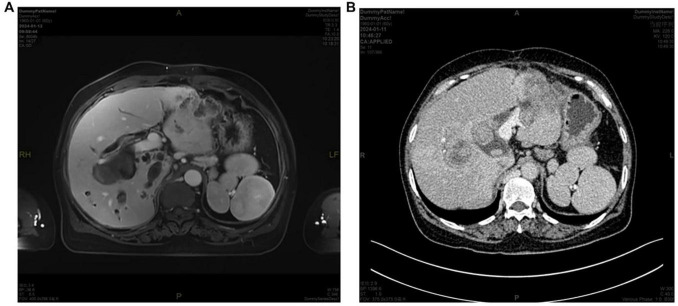
**(A)** MRI and **(B)** CT images demonstrating multiple spleens.

**FIGURE 3:**
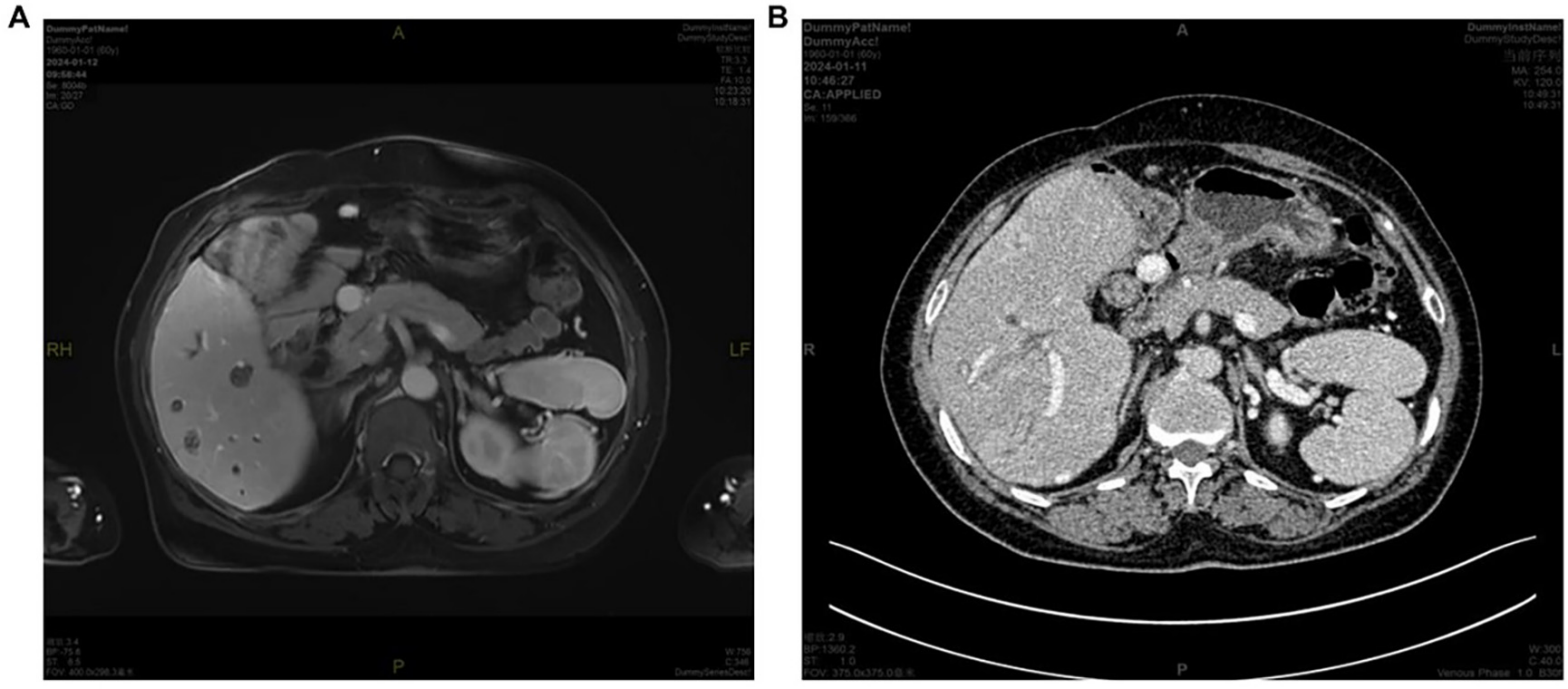
**(A)** MRI and **(B)** CT images demonstrating absence of the pancreatic tail.

**FIGURE 4:**
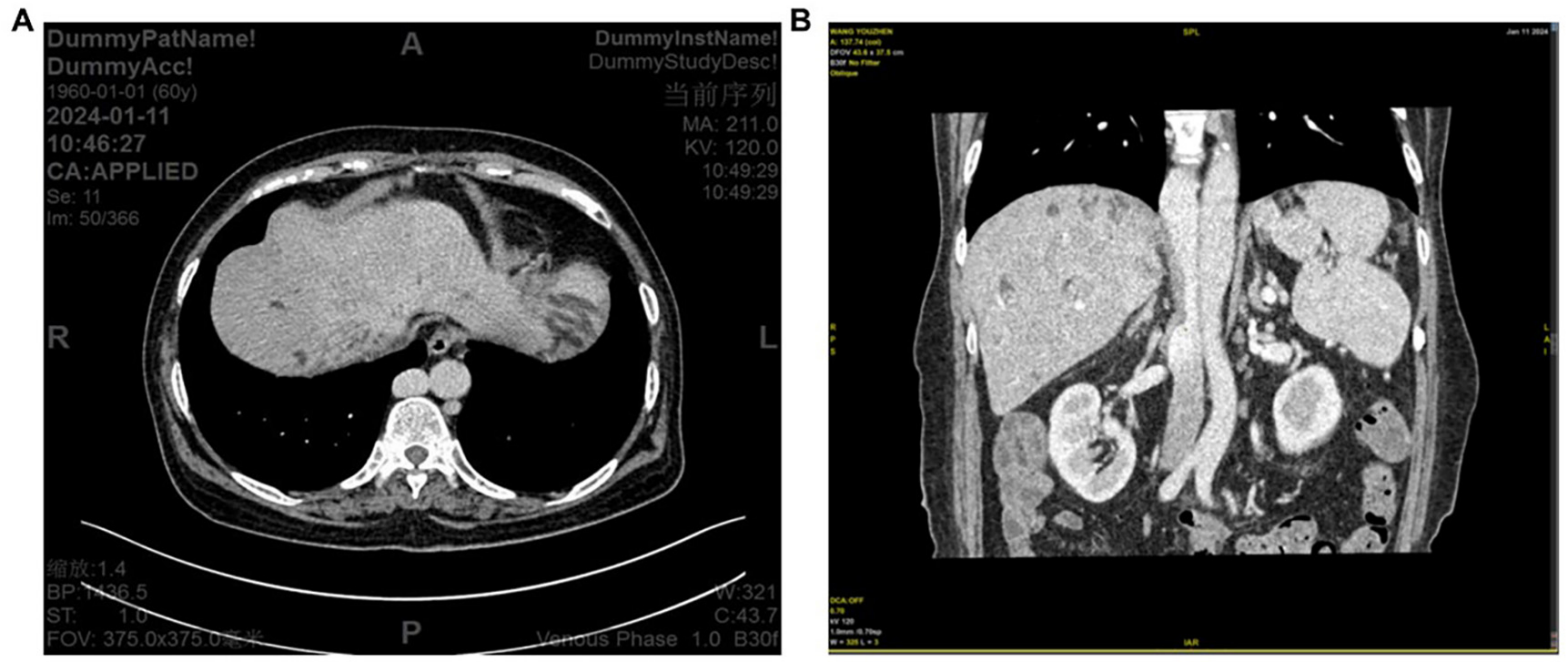
**(A)** Computed tomography (CT) image showing azygos vein dilation and tortuosity. **(B)** CT image showing focal non-visualization of the inferior vena cava (IVC).

A 60-years-old woman developed chills, fever (39.5 °C), nausea, vomiting, and mild upper abdominal discomfort and was admitted to our emergency department with a provisional diagnosis of intra- and extrahepatic biliary dilatation. Her medical history was notable for a cholecystectomy performed 20 years earlier. Laboratory tests revealed elevated inflammatory markers and a markedly increased carbohydrate antigen 19-9 (CA19-9) level (>1000 U/mL). Abdominal computed tomography (CT) scan revealed intrahepatic and extrahepatic biliary dilatation with multiple intrahepatic bile duct stones. Magnetic resonance cholangiopancreatography (MRCP) confirmed these findings (Figure 1) and additionally revealed several anomalies: multiple spleens consistent with polysplenia syndrome (Figure 2); absence of the pancreatic body and tail (Figure 3); and dilation and tortuosity of the azygos vein with focal absence of the superior segment of the inferior vena cava (Figure 4), suggesting developmental anomalies. Slightly enlarged lymph nodes were also observed in the abdominal cavity, retroperitoneum, and right cardiophrenic angle. Transthoracic echocardiography showed no cardiac abnormalities. Then she was considered in acute inflammatory state, the patient was initially managed with intravenous antibiotics. Following treatment, serial blood cultures remained negative, and inflammatory markers gradually normalized. Later, she was scheduled for surgical treatment.

The surgery was performed via the previous abdominal incision. Extensive intra-abdominal adhesions were encountered. The bile duct in the left lateral segment of the liver appeared dilated and contained multiple stones; on palpation, the segment was firm with indistinct margins from the anterior wall of the lesser curvature of the stomach. An incision into the duct yielded turbid bile and numerous stones. The surrounding tissue was inflamed and atrophic. The common bile duct (CBD) and intrahepatic ducts were also dilated (approximately 1.5 cm in diameter). The pancreatic tail was absent, and no additional visceral anomalies were observed. Subsequently, the left lateral segment of the liver was mobilized. The left lateral segmental hepatic artery, left portal vein branch, and left hepatic vein were ligated and divided, followed by transection of the left lateral lobe. The bile ducts on the left hepatic cut surface were opened, and additional calculi were removed. Choledochoscopy-guided CBD exploration and stone extraction were performed, and a T-tube was inserted for drainage. The transected left hepatic duct stump was sutured. The operation was completed successfully, and the patient was transferred to the ward in stable condition. Histopathological examination of the resected left lateral hepatic lobe confirmed the presence of multiple bile duct stones with foci of moderately to poorly differentiated cholangiocarcinoma identified within the bile ducts. No malignancy was detected in the remaining sections.

## Discussion

We report an adult case of polysplenia syndrome incidentally identified during the evaluation of intrahepatic bile duct stones. Polysplenia syndrome is a rare congenital condition characterized by multiple spleens and visceral and vascular abnormalities, and is typically diagnosed in infancy due to associated cardiac malformations (1, 3). However, in rare cases without severe cardiac involvement, the diagnosis may be delayed until adulthood, often uncovered during imaging for unrelated clinical conditions. Compared to other heterotaxy types, polysplenia syndrome shows a female predominance, a lower incidence and complexity of congenital heart disease than asplenia syndrome, and highly variable clinical presentations and prognosis.

During early embryonic development, the spleen and biliary system develop concurrently, and malformations in one may predispose to anomalies in the other (5). Specifically, splenic developmental defects, such as polysplenia or asplenia, are often associated with biliary maldevelopment. In infancy, these anomalies may present as biliary atresia (BA), a progressive cholangiopathy affecting the intrahepatic bile ducts, typically manifesting with neonatal jaundice and acholic stools (6). Approximately 10% of BA cases are reported to have concurrent polysplenia syndrome, highlighting a strong developmental link between the spleen and biliary system (7). In adults with polysplenia syndrome, biliary tract abnormalities often manifest as cholelithiasis or choledocholithiasis (8). The primary mechanism is believed to involve biliary stasis secondary to anatomical malformations, which facilitates bacterial colonization and stone formation mediated by β-glucuronidase activity (9). A previously reported case featured symmetric liver lobes, a midline gallbladder, and quadruple branching of intrahepatic ducts, which may have contributed to both gallbladder and bile duct stones (8). Our patient had a history of cholecystectomy for gallstones, possibly reflecting underlying biliary maldevelopment. The recurrence of bile duct stones further suggested persistent anatomical abnormalities. Admission MRI confirmed multiple intrahepatic bile duct stones, with dilation and mural thickening of the left hepatic duct and hilar bile ducts. While chronic obstruction may have altered the original biliary morphology, congenital structural anomalies likely represented the primary etiology for stone formation in this case.

Another factor underlying bile duct stone formation may be extrinsic compression of the CBD by adjacent organs or vessels due to aberrant visceral anatomy, which leads to biliary stasis. This may be induced by preduodenal portal vein (PDPV), a vascular anomaly frequently found in polysplenia syndrome (10–12); for example, it was identified in 7 of 8 cases in one series (13) and 7 of 19 cases in another (14). PDPV can compress the CBD, causing bile stasis and subsequent stone formation. Additionally, compression by malpositioned visceral organs can also impede bile flow (5). In our patient, multiple congenital anomalies, including polysplenia, non-visualization of the superior segment of the inferior vena cava (IVC), and a truncated pancreas, suggest abnormal visceral orientation, raising the possibility of extrinsic biliary compression. Moreover, the patient had previously undergone cholecystectomy, and no other common risk factors for choledocholithiasis [e.g., cirrhosis (9), chronic hemolysis, dietary or hormonal influences, or obesity (15)] were present. These findings support the hypothesis that persistent congenital biliary strictures and anatomical abnormalities contributed to the formation of bile duct stones in this case.

Clinical manifestations of polysplenia syndrome are highly variable and depend largely on the nature and severity of associated anomalies. In infancy, presentations are often dominated by severe congenital cardiac defects. Adult cases are rare and may present with splenic infarction (16), renal colic (2), nutcracker syndrome (17), and choledocholithiasis, with symptoms typically reflecting the involved organ system. Our patient presented with typical signs of cholangitis, including jaundice, abdominal pain, nausea, vomiting, and fever, suggesting that biliary complications may represent the predominant clinical manifestation in adult patients with polysplenia syndrome. This highlights the importance of maintaining a high level of suspicion for biliary tract abnormalities in patients with incidentally discovered polysplenia during health screening. Conversely, in patients with recurrent bile duct stones or atypical biliary anatomy, a diagnosis of polysplenia should be considered to avoid missed or delayed diagnosis.

Following polysplenia diagnosis, the evaluation and management of the case need to be carefully designed, particularly in surgical contexts. As a preventive measure, patients with incidentally diagnosed polysplenia should be proactively monitored and advised to mitigate the risk of cholelithiasis, such as dietary modifications to reduce cholesterol intake and glycemic control. In preoperative planning, thorough imaging and anatomical mapping should be performed. Surgeons should be alert to potential anomalies, such as PDPV (7), intestinal malrotation (5), and symmetric hepatic lobulation (8), which can increase the technical difficulty and risk of complications if unrecognized preoperatively. Notably, there are no standardized operative approaches for adult polysplenia syndrome complicated by cholelithiasis. In our case, we performed conventional procedures, including CBD exploration, choledocholithotomy, choledochoscopy, T-tube drainage, and partial hepatectomy. However, these interventions could not resolve the underlying congenital biliary strictures, potentially leaving a high risk of recurrence. Given the similarity between this pathophysiology and that of congenital choledochal cysts (18), whether biliary reconstruction (akin to cyst excision) could reduce recurrence in polysplenia remains an open question warranting further investigation.

## Conclusion

We reported a rare adult case of polysplenia syndrome complicated by multiple intrahepatic bile duct stones. While polysplenia syndrome is a rare congenital condition, its association with hepatobiliary malformations may predispose to chronic biliary stasis and infection. Our case highlights the importance of thorough pre-operational anatomical assessment, consideration of underlying structural abnormalities in surgical planning, and long-term monitoring for biliary complications.

## Data Availability

The datasets presented in this study can be found in online repositories. The names of the repository/repositories and accession number(s) can be found in this article/supplementary material.
